# Supplementary material for: No facilitatory effects of transcranial random noise stimulation on motion processing: A registered report
Source: bioRxiv. 2025 May 20:2025.03.18.643903. Preprint. [Version 4] doi: 10.1101/2025.03.18.643903 (PMC12132535; doi:10.1101/2025.03.18.643903)
Supplement: Supplement 1 [file NIHPP2025.03.18.643903v4-supplement-1.pdf]

bioRxiv preprint doi: https://doi.org/10.1101/2025.03.18.643903; this version posted May 20, 2025. The copyright holder for this preprint (which was not certified by peer review) is the author/funder. This article is a US Government work. It is not subject to copyright under 17 USC 105 and is also made available for use under a CC0 license.

Supplemental

Figure S1

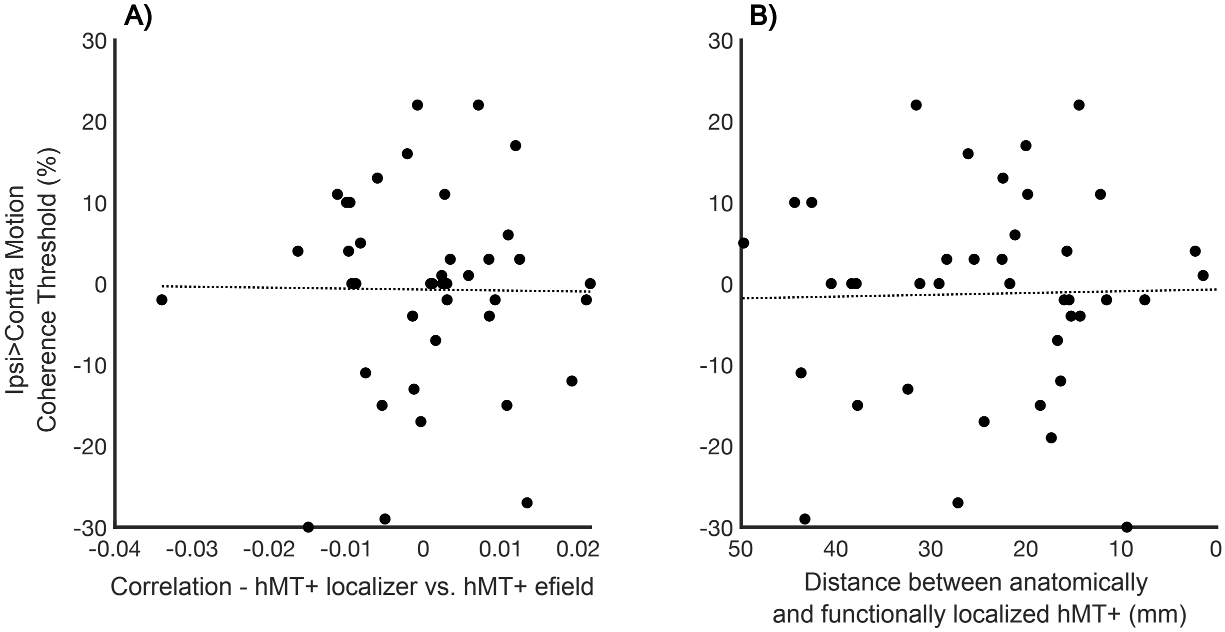

Supplemental Figure 1. *S1A) Correlation of functionally localized left hMT+ and e-field simulation used to predict ipsi>contralateral motion coherence threshold following hMT+ active stimulation. S1B) Distance between functionally localized left hMT+ and anatomically localized hMT+ correlated with ipsi>contralateral motion coherence threshold following hMT+ active stimulation. Least-squares line plotted.*

Table S1

Article	***Region***	***Task***
**Alvarez, R. P., et al., (2011)**	Middle/superior frontal gyrus	Fear conditioning
**Bereczkei, T., et al. (2013)**	Middle/superior frontal gyrus	Trust game
**Cheke, L. G., et al., (2017)**	Dorsolateral prefrontal gyrus (DLPFC)	What-where-when episodic memory task
**Collins, A. G. E., et al., (2017)**	Middle frontal gyrus	Reward learning
**Dove, A., et al. (2006)**	Left superior frontal gyrus	Memory encoding/retrieval
**FeldmanHall, O. et al. (2015)**	Left orbital frontal cortex	Pain vs. Gain task
**Forbes et al., (2012)**	Orbital frontal cortex	Spatial task
**Ford et al., (2011)**	Frontopolar prefrontal cortex	Autobiographical memory retrieval
**Greene et al., (2004)**	Middle Frontal Gyrus, DLPFC	Personal moral judgement
**Hartogsveld et al., (2018)**	Lateral frontal pole	Relational processing

bioRxiv preprint doi: https://doi.org/10.1101/2025.03.18.643903; this version posted May 20, 2025. The copyright holder for this preprint (which was not certified by peer review) is the author/funder. This article is a US Government work. It is not subject to copyright under 17 USC 105 and is also made available for use under a CC0 license.

**Hermans et al., (2008)**	Orbitofrontal cortex, BA47	Facial expression viewing
**Jenkins et al., (2014)**	Left middle frontal gyrus	Theory of mind task
**Kalpouzos et al., (2009)**	Left Frontal mid/mid orb/sup (10, 47)	Episodic memory encoding
**King et al., (2005)**	Anterior prefrontal cortex	Contextual episodic memory task
**Limb & Braun (2008)**	Polar medial prefrontal - middle	Piano improvisation
**Longe et al., (2009)**	Lateral PFC	Memory load
**Manelis et al., (2017)**	Frontal Pole	Temporal memory
**Olsson et al., (2007)**	Middle frontal gyrus	Social fear transmission
**Pyke et al., (2017)**	Brodmann Area 10	Typing in a keypad when either visuospatially trained or symbolically trained
**Schon et al., (2008)**	Left Frontomarginal sulcus (anterior VLPFC)	Delayed match to object or place
**Specht et al., (2009)**	Middle frontal gyrus	WCST
**Wendelken et al., (2008)**	Rostrolateral PFC	Delayed item recognition
**Winston et al., (2014)**	Left frontomarginal sulcus	Pain valuation task
**Wu et al., (2009)**	Middle frontal gyrus	Semantic task
**Zanto et al., (2016)**	Middle frontal orbital	Memory task with face stimuli

Supplemental Table 1. *Lists 25 of 203 articles associated with the MNI coordinates with the largest e-field value located in the frontal lobe (x=-27.61, y=56.15, z=1.22) from Neurosynth.org (NeuroSynth, RRID:SCR_006798)*

Appendix A: Simulations of stimulation montages

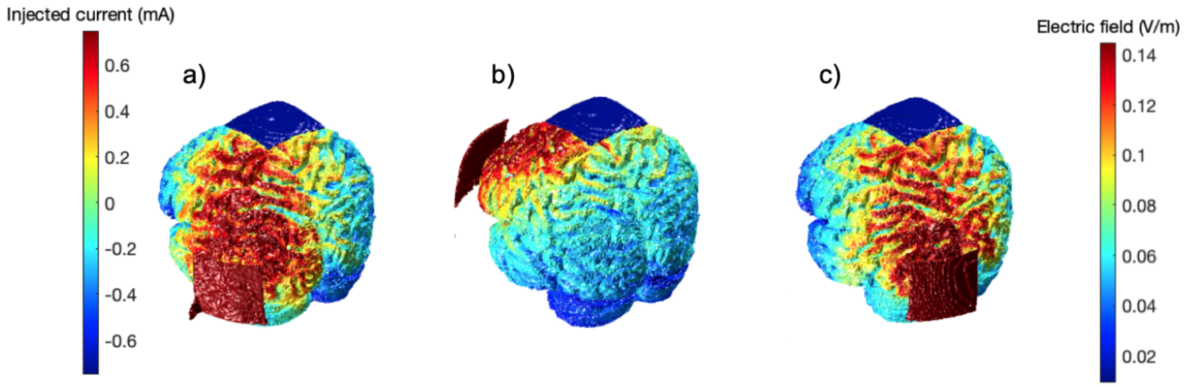

Appendix A, Figure 1: *Simulations of a) T5 (left hMT+) to Cz, b) Fp1 (left forehead) to Cz, c) O1 (left V1) to Cz using ROAST (Huang et al., 2019).*

bioRxiv preprint doi: https://doi.org/10.1101/2025.03.18.643903; this version posted May 20, 2025. The copyright holder for this preprint (which was not certified by peer review) is the author/funder. This article is a US Government work. It is not subject to copyright under 17 USC 105 and is also made available for use under a CC0 license.

The ROAST toolbox was designed for quasi-static modelling when the frequency is lower than 1k Hz. The head model is ohmic for the purpose of the simulation and is therefore the same for direct or alternating current (Huang et al., 2019).

Matlab Syntax:

Left hMT+ to Cz: roast('example/subject1.nii', {'T5',0.75,'Cz',-0.75}, 'electype', {'pad','pad'}, 'elecsize', {[50,50,3],[50,50,3]}, 'simulationTag', 'hMTsimulation')

Left forehead to Cz: roast('example/subject1.nii',{'Fp1',0.75,'Cz',-0.75}, 'electype',{'pad','pad'}, 'elecsize',{[50,50,3],[50,50,3]}, 'simulationTag', 'foreheadsimulation')

Left V1 to Cz: roast('example/subject1.nii',{'O1',0.75,'Cz',-0.75}, 'electype',{'pad','pad'}, 'elecsize',{[50,50,3],[50,50,3]}, 'simulationTag', 'V1simulation')

The active control of V1 to Cz as used by Ghin et al. (2018) was excluded from replication due to the overlap between the simulated electric field with the simulation of hMT+ to Cz. Overlap was quantified by analyzing the mutual information in the hMT+ and V1 simulations in comparison to the mutual information in the hMT+ and forehead simulations (Giangregorio, 2022). Where zero would denote complete independence between the simulations, and 2.50 complete dependence, hMT+ and V1 had a mutual information score of 1.30, whereas hMT+ and the forehead had a mutual information score of 0.95. Whilst some dependence is expected due to the same reference electrode position (Cz) in all montages, minimizing mutual information was preferable.

bioRxiv preprint doi: https://doi.org/10.1101/2025.03.18.643903; this version posted May 20, 2025. The copyright holder for this preprint (which was not certified by peer review) is the author/funder. This article is a US Government work. It is not subject to copyright under 17 USC 105 and is also made available for use under a CC0 license.

Appendix B: Power analyses

We performed three power analyses on our three comparisons of interest using a plot digitizer to extract the data from the figures:

First, we examined contralateral versus ipsilateral motion coherence thresholds following hf-tRNS to hMT+. Specifically, we used a mean of 28.75% related to the contralateral motion coherence threshold, a mean of 39.26% related to the ipsilateral motion coherence threshold, and standard deviation of 17.56% related to the standard deviation in the ipsilateral visual field following hf-tRNS targeted at left hMT+ (Ghin et al., 2018 Figure 2a; Cohen's d: 0.60). With a power of 0.9 and type I error rate of 2%, we calculated the need for 34 participants.
Second, we examined the difference between contralateral versus ipsilateral motion coherence thresholds following hMT+ targeted hf-tRNS versus the difference between contralateral versus ipsilateral motion coherence thresholds following sham hf-tRNS. We used a mean of 10.51% related to the difference in contralateral and ipsilateral motion coherence threshold for hMT+ targeted hf-tRNS, a mean of 2.59% related to the difference in contralateral and ipsilateral motion coherence threshold for hMT+ targeted sham tRNS, and standard deviation of 14.8% related to the standard deviation in the contralateral visual field following sham tRNS targeted at left hMT+ (Ghin et al., 2018 Figure 2a; Cohen's d: 0.54). With a power of 0.9 and type I error rate of 2%, we calculated the need for 42 participants. Finally, we examined the difference between contralateral versus ipsilateral motion coherence thresholds following hMT+ hf-tRNS versus the difference between contralateral versus ipsilateral motion coherence thresholds following forehead targeted hf-tRNS. We used a mean of 10.51% related to the difference in contralateral and ipsilateral motion coherence threshold for hMT+ targeted hf-tRNS, a mean of 1.17% related to the difference in contralateral and ipsilateral motion coherence threshold for forehead targeted hf-tRNS, and standard deviation of 8.44% related to the standard deviation in the contralateral visual field following hf- tRNS targeted at the left forehead (Ghin et al., 2018

bioRxiv preprint doi: https://doi.org/10.1101/2025.03.18.643903; this version posted May 20, 2025. The copyright holder for this preprint (which was not certified by peer review) is the author/funder. This article is a US Government work. It is not subject to copyright under 17 USC 105 and is also made available for use under a CC0 license.

Figures 2a and 4a; Cohen's d: 1.11). With a power of 0.9 and type I error rate of 2%, we calculated the need for 12 participants.

To maximally power our study for our three effect sizes of interest, we collected 42 participants.

Appendix C: Screening participants for 70% motion discrimination

During piloting (see below), we were unable to establish a threshold at 70% correct on some participants due to poor performance. A threshold target of 70% is necessary for the maximum likelihood procedure thresholding to function. In order to replicate the original findings, rather than reduce the p-target, we screened participants using a non-adaptive constant thresholding procedure.

Piloting the RDKs with non-adaptive constant thresholding

Using the same random dot kinematograms (RDKs) presented in motion discrimination task as described in the Stimuli section of the Method, we presented eight blocks of RDKs (n trials = 64) at a range of motion coherence levels from 25% to 100% in steps of 5%. This resulted in 32 trials per motion coherence level across all blocks. Using these data, we fit a psychometric function using a logit glm, and find the motion coherence at which participants were performing at 70% correct.

bioRxiv preprint doi: https://doi.org/10.1101/2025.03.18.643903; this version posted May 20, 2025. The copyright holder for this preprint (which was not certified by peer review) is the author/funder. This article is a US Government work. It is not subject to copyright under 17 USC 105 and is also made available for use under a CC0 license.

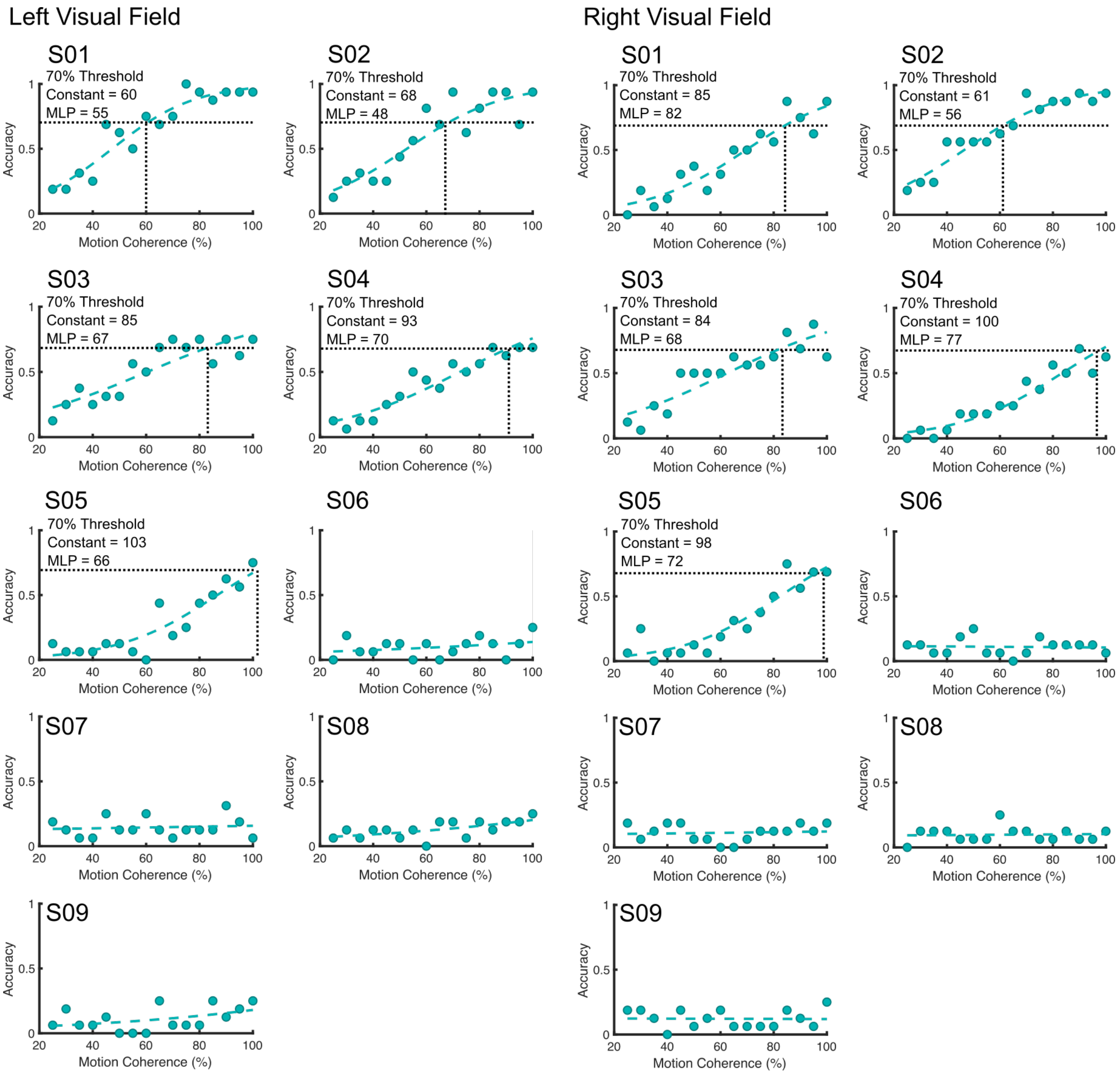

Appendix C, Figure 1: *Pilot data of nine participants in left and right visual fields. Data from the constant thresholding plotted with a psychometric function and MLP reported.*

We plot these functions for nine pilot participants in Appendix C, Figure 1. Five performed the motion discrimination task above 70% at one or more of the motion coherence levels, the other four performed below 70% at all motion coherence levels.

bioRxiv preprint doi: https://doi.org/10.1101/2025.03.18.643903; this version posted May 20, 2025. The copyright holder for this preprint (which was not certified by peer review) is the author/funder. This article is a US Government work. It is not subject to copyright under 17 USC 105 and is also made available for use under a CC0 license.

The five pilot participants able to perform the task above 70% correct returned for a second session using the MLP, and we found the MLP provided estimated thresholds lower than the constant thresholding performed in the first session, which may reflect a session effect of learning or increased sensitivity of the MLP approach.

Screened participant data

In Appendix C, Figure 2 we present all screened participants (1-42 included in the study; 43-87 excluded). Participants were excluded due to subthreshold performance at all motion coherence levels.

bioRxiv preprint doi: https://doi.org/10.1101/2025.03.18.643903; this version posted May 20, 2025. The copyright holder for this preprint (which was not certified by peer review) is the author/funder. This article is a US Government work. It is not subject to copyright under 17 USC 105 and is also made available for use under a CC0 license.

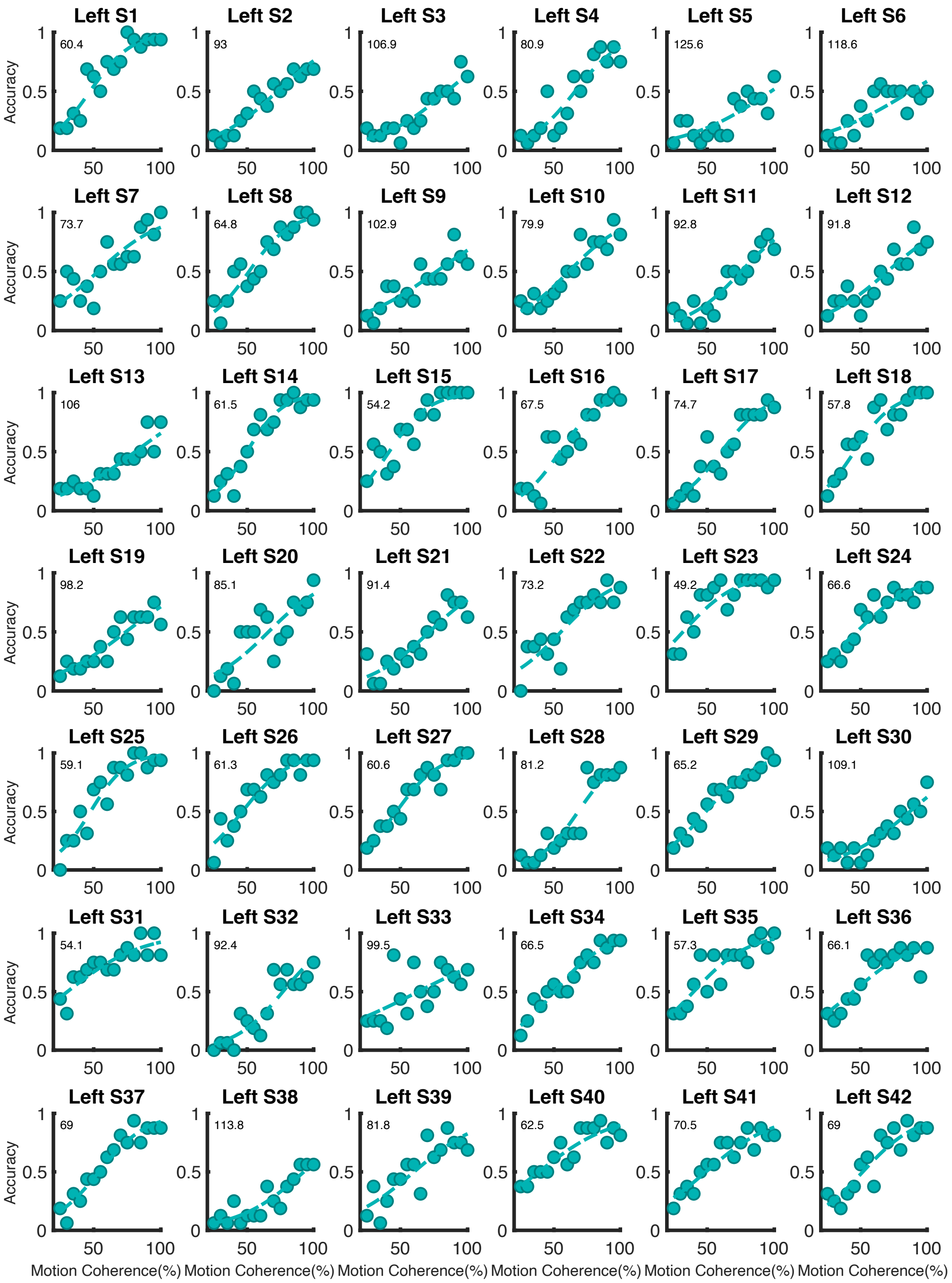

bioRxiv preprint doi: https://doi.org/10.1101/2025.03.18.643903; this version posted May 20, 2025. The copyright holder for this preprint (which was not certified by peer review) is the author/funder. This article is a US Government work. It is not subject to copyright under 17 USC 105 and is also made available for use under a CC0 license.

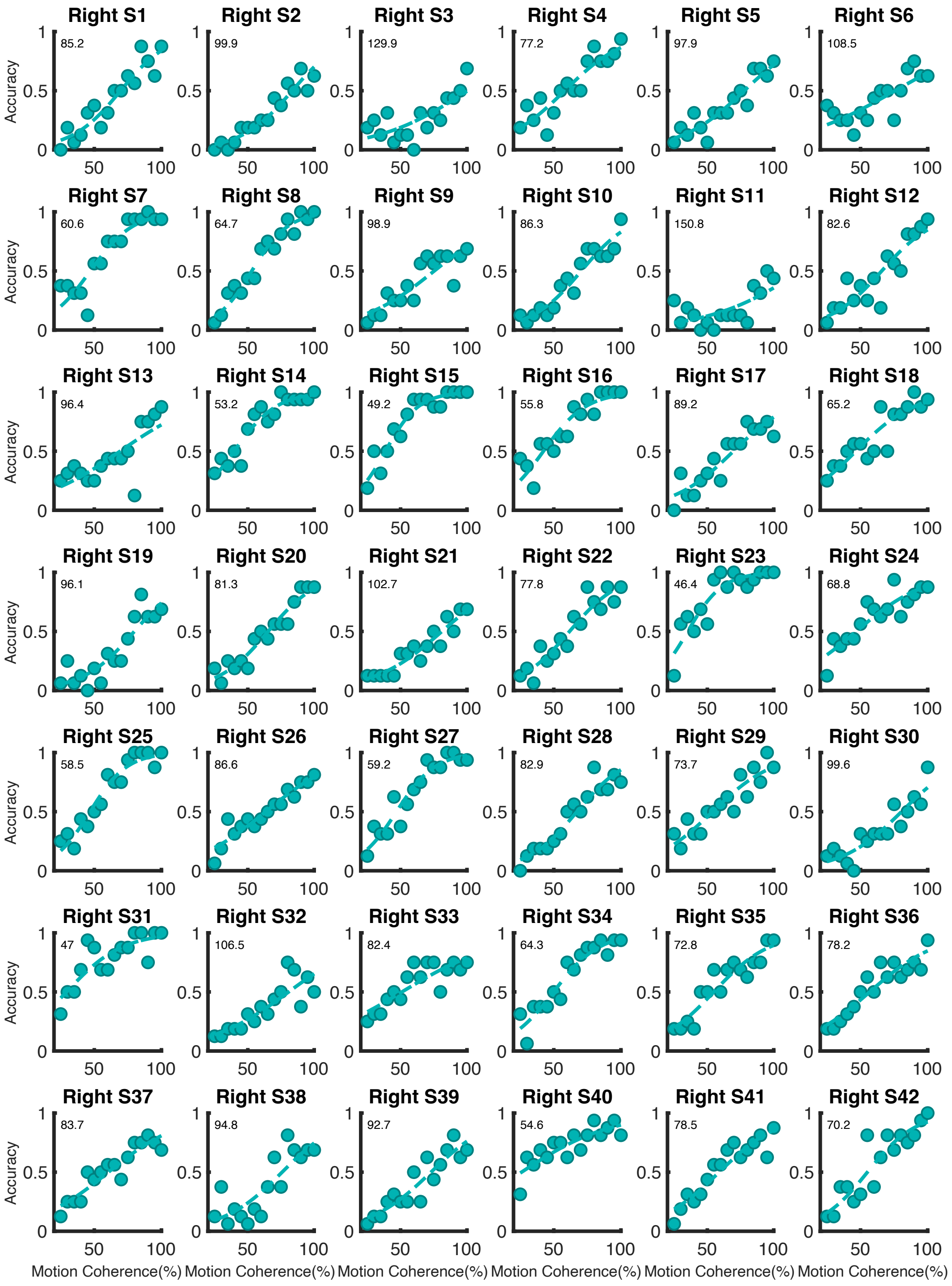

bioRxiv preprint doi: https://doi.org/10.1101/2025.03.18.643903; this version posted May 20, 2025. The copyright holder for this preprint (which was not certified by peer review) is the author/funder. This article is a US Government work. It is not subject to copyright under 17 USC 105 and is also made available for use under a CC0 license.

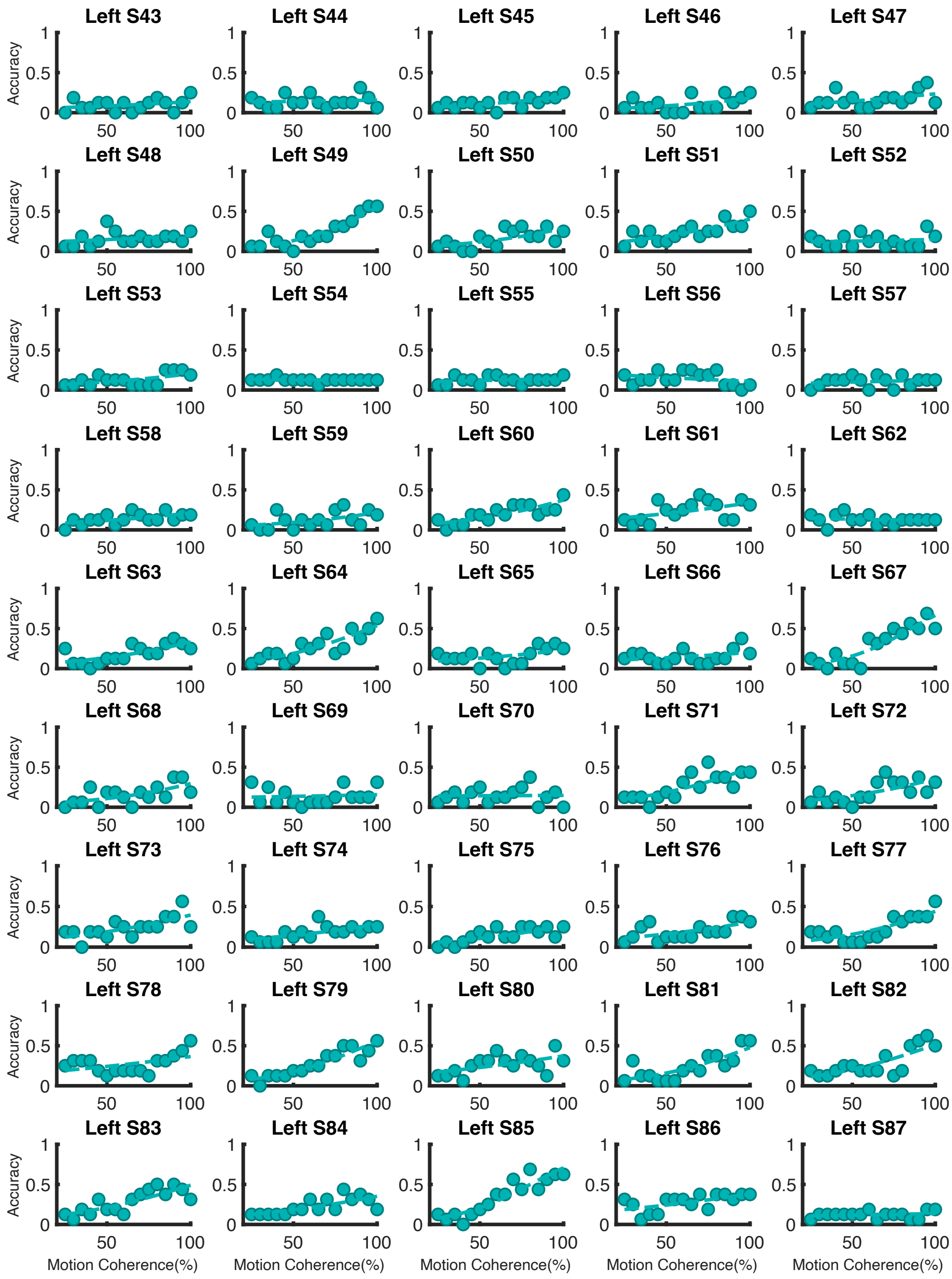

bioRxiv preprint doi: https://doi.org/10.1101/2025.03.18.643903; this version posted May 20, 2025. The copyright holder for this preprint (which was not certified by peer review) is the author/funder. This article is a US Government work. It is not subject to copyright under 17 USC 105 and is also made available for use under a CC0 license.

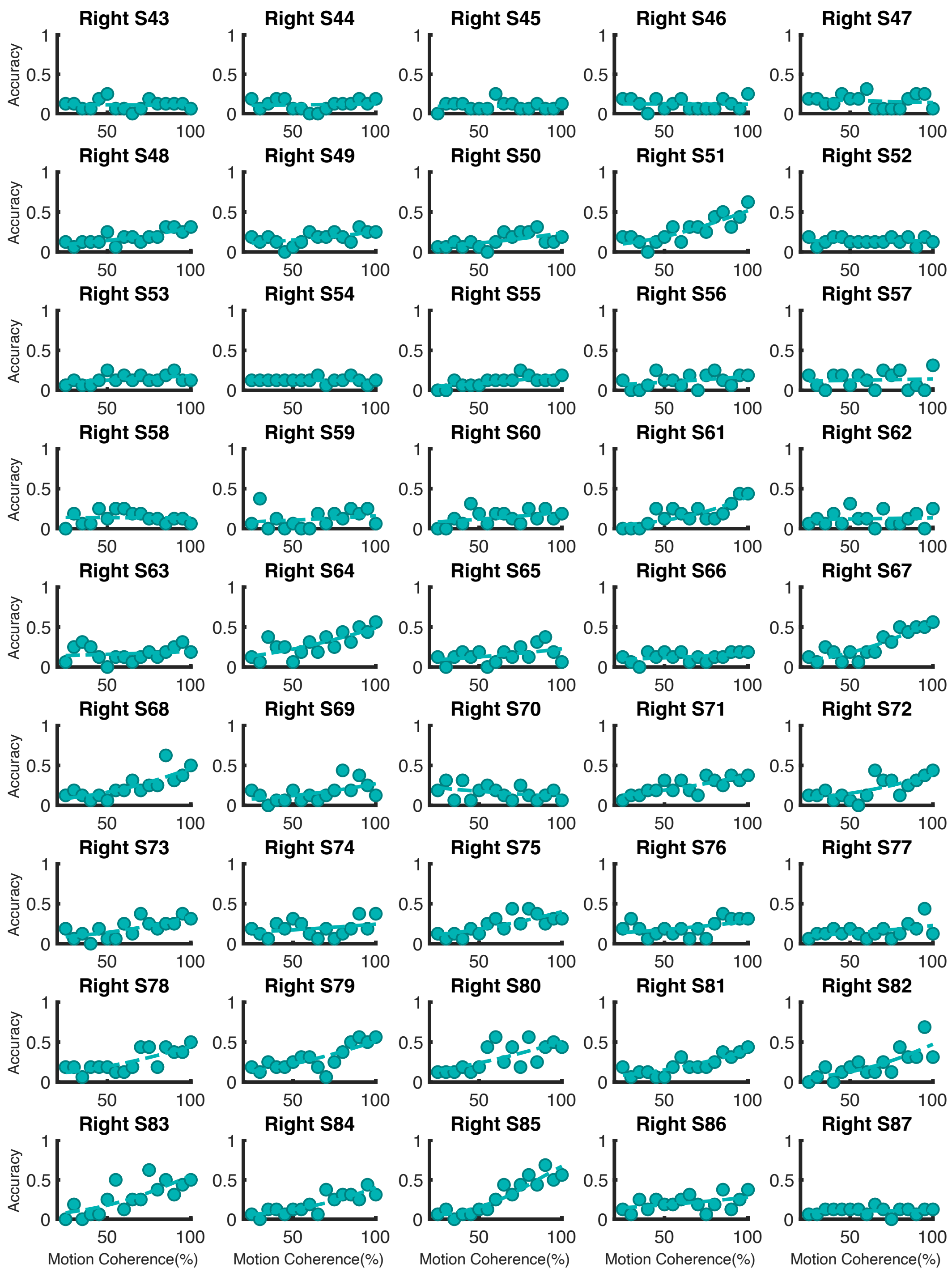

bioRxiv preprint doi: https://doi.org/10.1101/2025.03.18.643903; this version posted May 20, 2025. The copyright holder for this preprint (which was not certified by peer review) is the author/funder. This article is a US Government work. It is not subject to copyright under 17 USC 105 and is also made available for use under a CC0 license.

Appendix C, Figure 2: *Screening data of 87 participants in left and right visual fields. Data from the constant thresholding plotted with a psychometric function and constant threshold reported for participants 1-42 who were included in the main study.*

References

Ajina, Sara, Christopher Kennard, Geraint Rees, and Holly Bridge. 2015. "Motion Area V5/MT+ Response to Global Motion in the Absence of V1 Resembles Early Visual Cortex." *Brain* 138 (1): 164–78. https://doi.org/10.1093/brain/awu328.

Alvarez, R. P., Chen, G., Bodurka, J., Kaplan, R., & Grillon, C. (2011). Phasic and sustained fear in humans elicits distinct patterns of brain activity. *NeuroImage*, *55*(1), 389–400. https://doi.org/10.1016/j.neuroimage.2010.11.057

Amano, K., Wandell, B. A., & Dumoulin, S. O. (2009). Visual Field Maps, Population Receptive Field Sizes, and Visual Field Coverage in the Human MT+ Complex. *Journal of Neurophysiology*, *102*(5), 2704–2718. https://doi.org/10.1152/jn.00102.2009

Antal, Andrea, Gyula Kovács, Leila Chaieb, Csaba Cziraki, Walter Paulus, and Mark W. Greenlee. 2012. "Cathodal Stimulation of Human MT+ Leads to Elevated FMRI Signal: A TDCS-FMRI Study." *Restorative Neurology and Neuroscience* 30 (3): 255–63. https://doi.org/10.3233/RNN-2012-110208.

Antal, Andrea, Michael A. Nitsche, Wolfgang Kruse, Tamás Z. Kincses, Klaus-Peter Hoffmann, and Walter Paulus. 2004. "Direct Current Stimulation over V5 Enhances Visuomotor Coordination by Improving Motion Perception in Humans." *Journal of Cognitive Neuroscience* 16 (4): 521–27. https://doi.org/10.1162/089892904323057263.

Battaglini, Luca, Stefano Noventa, and Clara Casco. 2017. "Anodal and Cathodal Electrical Stimulation over V5 Improves Motion Perception by Signal Enhancement and Noise Reduction." *Brain Stimulation* 10 (4): 773–79. https://doi.org/10.1016/j.brs.2017.04.128.

Bereczkei, T., Deak, A., Papp, P., Perlaki, G., & Orsi, G. (2013). Neural correlates of Machiavellian strategies in a social dilemma task. *Brain and Cognition*, *82*(1), 108–116. https://doi.org/10.1016/j.bandc.2013.02.012

Braddick, Oliver J, Justin M D O'Brien, John Wattam-Bell, Janette Atkinson, Tom Hartley, and Robert Turner. 2001. "Brain Areas Sensitive to Coherent Visual Motion." *Perception* 30 (1): 61–72. https://doi.org/10.1068/p3048.

Brem, Anna-Katharine, Jessamy Norton-Ford Almquist, Karen Mansfield, Franziska Plessow, Francesco Sella, Emiliano Santarnecchi, Umut Orhan, et al. 2018. "Modulating Fluid Intelligence Performance through Combined Cognitive Training and Brain Stimulation." *Neuropsychologia* 118 (September): 107–14. https://doi.org/10.1016/j.neuropsychologia.2018.04.008.

Campana, G., Cowey, A., & Walsh, V. (2002). Priming of Motion Direction and Area V5/MT: A Test of Perceptual Memory. *Cerebral Cortex*, *12*(6), 663–669. https://doi.org/10.1093/cercor/12.6.663

Campana, G., Cowey, A., & Walsh, V. (2006). Visual Area V5/MT Remembers "What" but Not "Where." *Cerebral Cortex*, *16*(12), 1766–1770. https://doi.org/10.1093/cercor/bhj111

Campana, Gianluca, Rebecca Camilleri, Beatrice Moret, Filippo Ghin, and Andrea Pavan. 2016. "Opposite Effects of High- and Low-Frequency Transcranial Random Noise Stimulation Probed with Visual Motion Adaptation." *Scientific Reports* 6 (1): 38919. https://doi.org/10.1038/srep38919.

bioRxiv preprint doi: https://doi.org/10.1101/2025.03.18.643903; this version posted May 20, 2025. The copyright holder for this preprint (which was not certified by peer review) is the author/funder. This article is a US Government work. It is not subject to copyright under 17 USC 105 and is also made available for use under a CC0 license.

Campana, Gianluca, Marcello Maniglia, and Andrea Pavan. 2013. “Common (and Multiple) Neural Substrates for Static and Dynamic Motion after-Effects: A RTMS Investigation.” *Cortex* 49 (9): 2590–94. https://doi.org/10.1016/j.cortex.2013.07.001.

Cappelletti, M., E. Gessaroli, R. Hithersay, M. Mitolo, D. Didino, R. Kanai, R. Cohen Kadosh, and V. Walsh. 2013. “Transfer of Cognitive Training across Magnitude Dimensions Achieved with Concurrent Brain Stimulation of the Parietal Lobe.” *Journal of Neuroscience* 33 (37): 14899–907. https://doi.org/10.1523/JNEUROSCI.1692-13.2013.

Chakraborty, Arijit, Tiffany T. Tran, Andrew E. Silva, Deborah Giaschi, and Benjamin Thompson. 2021. “Continuous Theta Burst TMS of Area MT+ Impairs Attentive Motion Tracking.” *European Journal of Neuroscience* 54 (9): 7289–7300. https://doi.org/10.1111/ejn.15480.

Cheke, L. G., Bonnici, H. M., Clayton, N. S., & Simons, J. S. (2017). Obesity and insulin resistance are associated with reduced activity in core memory regions of the brain. *Neuropsychologia*, *96*, 137–149. https://doi.org/10.1016/j.neuropsychologia.2017.01.013

Collins, A. G. E., Ciullo, B., Frank, M. J., & Badre, D. (2017). Working Memory Load Strengthens Reward Prediction Errors. *The Journal of Neuroscience: The Official Journal of the Society for Neuroscience*, *37*(16), 4332–4342. https://doi.org/10.1523/JNEUROSCI.2700-16.2017

Contò, Federica, Grace Edwards, Sarah Tyler, Danielle Parrott, Emily Grossman, and Lorella Battelli. 2021. “Attention Network Modulation via TRNS Correlates with Attention Gain.” Edited by Taraz Lee, Richard B Ivry, and Taraz Lee. *ELife* 10 (November): e63782. https://doi.org/10.7554/eLife.63782.

Cox, Robert W. 1996. “AFNI: Software for Analysis and Visualization of Functional Magnetic Resonance Neuroimages.” *Computers and Biomedical Research* 29 (3): 162–73. https://doi.org/10.1006/cbmr.1996.0014.

Dove, A., Brett, M., Cusack, R., & Owen, A. M. (2006). Dissociable contributions of the mid-ventrolateral frontal cortex and the medial temporal lobe system to human memory. *NeuroImage*, *31*(4), 1790–1801. https://doi.org/10.1016/j.neuroimage.2006.02.035

FeldmanHall, O., Dalgleish, T., Evans, D., & Mobbs, D. (2015). Empathic concern drives costly altruism. *NeuroImage*, *105*, 347–356. https://doi.org/10.1016/j.neuroimage.2014.10.043

Forbes, C. E., Cox, C. L., Schmader, T., & Ryan, L. (2012). Negative stereotype activation alters interaction between neural correlates of arousal, inhibition and cognitive control. *Social Cognitive and Affective Neuroscience*, *7*(7), 771–781. https://doi.org/10.1093/scan/nsr052

Ford, J. H., Addis, D. R., & Giovanello, K. S. (2011). Differential neural activity during search of specific and general autobiographical memories elicited by musical cues. *Neuropsychologia*, *49*(9), 2514–2526. https://doi.org/10.1016/j.neuropsychologia.2011.04.032

Greene, J. D., Nystrom, L. E., Engell, A. D., Darley, J. M., & Cohen, J. D. (2004). The Neural Bases of Cognitive Conflict and Control in Moral Judgment. *Neuron*, *44*(2), 389–400. https://doi.org/10.1016/j.neuron.2004.09.027

Ghin, Filippo, Andrea Pavan, Adriano Contillo, and George Mather. 2018. “The Effects of High-Frequency Transcranial Random Noise Stimulation (Hf-TRNS) on Global Motion Processing: An Equivalent Noise Approach.” *Brain Stimulation* 11 (6): 1263–75. https://doi.org/10.1016/j.brs.2018.07.048.

Giangregorio (2022). Very fast Mutual Information betweentwo images. (https://www.mathworks.com/matlabcentral/fileexchange/36538-very-fast-mutual-information-betweentwo-images), MATLAB Central File Exchange. Retrieved September 7, 2022.

bioRxiv preprint doi: https://doi.org/10.1101/2025.03.18.643903; this version posted May 20, 2025. The copyright holder for this preprint (which was not certified by peer review) is the author/funder. This article is a US Government work. It is not subject to copyright under 17 USC 105 and is also made available for use under a CC0 license.

Grassi, Massimo, and Alessandro Soranzo. 2009. "MLP: A MATLAB Toolbox for Rapid and Reliable Auditory Threshold Estimation." *Behavior Research Methods* 41 (1): 20–28. https://doi.org/10.3758/BRM.41.1.20.

Greenwald, A. G. (1976). Within-subjects designs: To use or not to use? *Psychological Bulletin*, *83*(2), 314–320. https://doi.org/10.1037/0033-2909.83.2.314

Groen, O. van der, & Wenderoth, N. (2016). Transcranial Random Noise Stimulation of Visual Cortex: Stochastic Resonance Enhances Central Mechanisms of Perception. *Journal of Neuroscience*, *36*(19), 5289–5298. https://doi.org/10.1523/JNEUROSCI.4519-15.2016

Groen, Onno van der, Weronika Potok, Nicole Wenderoth, Grace Edwards, Jason B. Mattingley, and Dylan Edwards. 2022. "Using Noise for the Better: The Effects of Transcranial Random Noise Stimulation on the Brain and Behavior." *Neuroscience & Biobehavioral Reviews* 138 (July): 104702. https://doi.org/10.1016/j.neubiorev.2022.104702.

Hartogsveld, B., Bramson, B., Vijayakumar, S., van Campen, A. D., Marques, J. P., Roelofs, K., Toni, I., Bekkering, H., & Mars, R. B. (2018). Lateral frontal pole and relational processing: Activation patterns and connectivity profile. *Behavioural Brain Research*, *355*, 2–11. https://doi.org/10.1016/j.bbr.2017.08.003

Hermans, E. J., Ramsey, N. F., & van Honk, J. (2008). Exogenous testosterone enhances responsiveness to social threat in the neural circuitry of social aggression in humans. *Biological Psychiatry*, *63*(3), 263–270. https://doi.org/10.1016/j.biopsych.2007.05.013

Herpich, Florian, Michael D. Melnick, Sara Agosta, Krystel R. Huxlin, Duje Tadin, and Lorella Battelli. 2019. "Boosting Learning Efficacy with Noninvasive Brain Stimulation in Intact and Brain-Damaged Humans." *The Journal of Neuroscience* 39 (28): 5551–61. https://doi.org/10.1523/JNEUROSCI.3248-18.2019.

Huang, Yu, Abhishek Datta, Marom Bikson, and Lucas C Parra. 2019. "Realistic Volumetric-Approach to Simulate Transcranial Electric Stimulation—ROAST— a Fully Automated Open-Source Pipeline." *J. Neural Eng.*, 16.

Jenkins, A. C., Dodell-Feder, D., Saxe, R., & Knobe, J. (2014). The neural bases of directed and spontaneous mental state attributions to group agents. *PloS One*, *9*(8), e105341. https://doi.org/10.1371/journal.pone.0105341

Kalpouzos, G., Chételat, G., Landeau, B., Clochon, P., Viader, F., Eustache, F., & Desgranges, B. (2009). Structural and metabolic correlates of episodic memory in relation to the depth of encoding in normal aging. *Journal of Cognitive Neuroscience*, *21*(2), 372–389. https://doi.org/10.1162/jocn.2008.21027

King, J. A., Hartley, T., Spiers, H. J., Maguire, E. A., & Burgess, N. (2005). Anterior prefrontal involvement in episodic retrieval reflects contextual interference. *NeuroImage*, *28*(1), 256–267. https://doi.org/10.1016/j.neuroimage.2005.05.057

Laycock, Robin, David P. Crewther, Paul B. Fitzgerald, and Sheila G. Crewther. 2007. "Evidence for Fast Signals and Later Processing in Human V1/V2 and V5/MT+: A TMS Study of Motion Perception." *Journal of Neurophysiology* 98 (3): 1253–62. https://doi.org/10.1152/jn.00416.2007.

Limb, C. J., & Braun, A. R. (2008). Neural Substrates of Spontaneous Musical Performance: An fMRI Study of Jazz Improvisation. *PLOS ONE*, *3*(2), e1679. https://doi.org/10.1371/journal.pone.0001679

Longe, O., Senior, C., & Rippon, G. (2009). The lateral and ventromedial prefrontal cortex work as a dynamic integrated system: Evidence from FMRI connectivity analysis. *Journal of Cognitive Neuroscience*, *21*(1), 141–154. https://doi.org/10.1162/jocn.2009.21012

Manelis, A., Popov, V., Paynter, C., Walsh, M., Wheeler, M. E., Vogt, K. M., & Reder, L. M. (2017). Cortical Networks Involved in Memory for Temporal Order. *Journal of Cognitive Neuroscience*, *29*(7), 1253–1266. https://doi.org/10.1162/jocn_a_01123

McKeefry, D. J., M. P. Burton, C. Vakrou, B. T. Barrett, and A. B. Morland. 2008. "Induced Deficits in Speed Perception by Transcranial Magnetic Stimulation of Human Cortical

bioRxiv preprint doi: https://doi.org/10.1101/2025.03.18.643903; this version posted May 20, 2025. The copyright holder for this preprint (which was not certified by peer review) is the author/funder. This article is a US Government work. It is not subject to copyright under 17 USC 105 and is also made available for use under a CC0 license.

Areas V5/MT+ and V3A." *Journal of Neuroscience* 28 (27): 6848–57. https://doi.org/10.1523/JNEUROSCI.1287-08.2008.

Olsson, A., Nearing, K. I., & Phelps, E. A. (2007). Learning fears by observing others: The neural systems of social fear transmission. *Social Cognitive and Affective Neuroscience*, *2*(1), 3–11. https://doi.org/10.1093/scan/nsm005

Pavan, Andrea, Filippo Ghin, Adriano Contillo, Chiara Milesi, Gianluca Campana, and George Mather. 2019. "Modulatory Mechanisms Underlying High-Frequency Transcranial Random Noise Stimulation (Hf-TRNS): A Combined Stochastic Resonance and Equivalent Noise Approach." *Brain Stimulation* 12 (4): 967–77. https://doi.org/10.1016/j.brs.2019.02.018.

Pyke, A. A., Fincham, J. M., & Anderson, J. R. (2017). When math operations have visuospatial meanings versus purely symbolic definitions: Which solving stages and brain regions are affected? *NeuroImage*, *153*, 319–335. https://doi.org/10.1016/j.neuroimage.2017.03.046

Romanska, Aleksandra, Constantin Rezlescu, Tirta Susilo, Bradley Duchaine, and Michael J. Banissy. 2015. "High-Frequency Transcranial Random Noise Stimulation Enhances Perception of Facial Identity." *Cerebral Cortex* 25 (11): 4334–40. https://doi.org/10.1093/cercor/bhv016.

Schon, K., Tinaz, S., Somers, D. C., & Stern, C. E. (2008). Delayed match to object or place: An event-related fMRI study of short-term stimulus maintenance and the role of stimulus pre-exposure. *NeuroImage*, *39*(2), 857–872. https://doi.org/10.1016/j.neuroimage.2007.09.023

Snowball, Albert, Ilias Tachtsidis, Tudor Popescu, Jacqueline Thompson, Margarete Delazer, Laura Zamarian, Tingting Zhu, and Roi Cohen Kadosh. 2013. "Long-Term Enhancement of Brain Function and Cognition Using Cognitive Training and Brain Stimulation." *Current Biology* 23 (11): 987–92. https://doi.org/10.1016/j.cub.2013.04.045.

Specht, K., Lie, C.-H., Shah, N. J., & Fink, G. R. (2009). Disentangling the prefrontal network for rule selection by means of a non-verbal variant of the Wisconsin Card Sorting Test. *Human Brain Mapping*, *30*(5), 1734–1743. https://doi.org/10.1002/hbm.20637

Strong, Samantha L., Edward H. Silson, André D. Gouws, Antony B. Morland, and Declan J. McKeefry. 2017. "A Direct Demonstration of Functional Differences between Subdivisions of Human V5/MT+." *Cerebral Cortex* 27 (1): 1–10. https://doi.org/10.1093/cercor/bhw362.

Terney, D., L. Chaieb, V. Moliadze, A. Antal, and W. Paulus. 2008. "Increasing Human Brain Excitability by Transcranial High-Frequency Random Noise Stimulation." *Journal of Neuroscience* 28 (52): 14147–55. https://doi.org/10.1523/JNEUROSCI.4248-08.2008.

Wendelken, C., Bunge, S. A., & Carter, C. S. (2008). Maintaining structured information: An investigation into functions of parietal and lateral prefrontal cortices. *Neuropsychologia*, *46*(2), 665–678. https://doi.org/10.1016/j.neuropsychologia.2007.09.015

Westwood, Samuel James. 2020. "Investigating Cognitive and Therapeutic Effects of Transcranial Electric Stimulation (TES): A Short Guide for Reproducible and Transparent Research." Preprint. PsyArXiv. https://doi.org/10.31234/osf.io/8qms2.

Willis, Megan L., Andrea I. Costantino, Michael. A. Nitsche, Romina Palermo, and Davide Rivolta. 2019. "Anodal TDCS and High-Frequency TRNS Targeting the Occipitotemporal Cortex Do Not Always Enhance Face Perception." *Frontiers in Neuroscience* 13: 78. https://doi.org/10.3389/fnins.2019.00078.

Winston, J. S., Vlaev, I., Seymour, B., Chater, N., & Dolan, R. J. (2014). Relative Valuation of Pain in Human Orbitofrontal Cortex. *Journal of Neuroscience*, *34*(44), 14526–14535. https://doi.org/10.1523/JNEUROSCI.1706-14.2014

Wu, X., Lu, J., Chen, K., Long, Z., Wang, X., Shu, H., Li, K., Liu, Y., & Yao, L. (2009). Multiple neural networks supporting a semantic task: An fMRI study using independent

bioRxiv preprint doi: https://doi.org/10.1101/2025.03.18.643903; this version posted May 20, 2025. The copyright holder for this preprint (which was not certified by peer review) is the author/funder. This article is a US Government work. It is not subject to copyright under 17 USC 105 and is also made available for use under a CC0 license.

component analysis. *NeuroImage*, *45*(4), 1347–1358. https://doi.org/10.1016/j.neuroimage.2008.12.050

Zanto, T. P., Clapp, W. C., Rubens, M. T., Karlsson, J., & Gazzaley, A. (2016). Expectations of Task Demands Dissociate Working Memory and Long-Term Memory Systems. *Cerebral Cortex (New York, NY)*, *26*(3), 1176–1186. https://doi.org/10.1093/cercor/bhu307
